# Organizational factors and patient outcomes in Brazilian ICUs: the ORCHESTRA study

**DOI:** 10.1186/cc14580

**Published:** 2015-03-16

**Authors:** M Soares, JM Kahn, FA Bozza, T Lisboa, LP Azevedo, W Viana, L Brauer, PE Brasil, DC Angus, JI Salluh

**Affiliations:** 1 DOr Institute for Research and Education - IDOR, Rio De Janeiro, Brazil; 2 University of Pittsburgh Medical Center, Pittsburgh, PA, USA; 3 Sta. Casa de Porto Alegre, Brazil; 4 Hospital Sírio Libanês, São Paulo, Brazil; 5 Hospital Copa DOr, Rio de Janeiro, Brazil; 6 Hospital Sao Luiz Itaim, São Paulo, Brazil

## Introduction

The aim was to investigate the impact of organizational factors on patient outcomes in a large sample of Brazilian ICUs.

## Methods

A retrospective cohort study of 59,483 patients admitted to 78 ICUs in 51 hospitals during 2013. We retrieved demographic, clinical and outcome data from an electronic ICU quality registry (Epimed Monitor System). We surveyed ICUs using a standardized questionnaire regarding hospital and ICU structure, organization, staffing patterns, process of care, and family care policies. We used multilevel logistic regression analysis to identify characteristics associated with hospital mortality.

## Results

ICUs were mostly medical or medical-surgical (62.79%) and located in private hospitals (67.86%). Approximately half (40.51%) had critical care training programs. Median physician and nurse staff - bed ratios were 0.15 (IQR, 0.12 to 0.19) and 0.71 (0.61 to 0.84); board-certified intensivists were present 24/7 in 16 (21%) of ICUs. Routine clinical rounds occurred in 67 (86%) and daily clinical checklists were used in 36 (46%) ICUs. Most frequently implemented protocols focused on sepsis management and VAP and CLABSI prevention. Median number of patients per center was 898 (IQR 585 to 1,715) and there were 67% medical admissions; 18% patients received mechanical ventilation (MV). Median SAPS 3 score was 41 (33 to 52) points. ICU and hospital mortality rates were 9.6% and 14.3%, respectively. Adjusting for relevant patients' characteristics (SAPS 3 score, diagnostic admission category, chronic health status, comorbidities, MV use), case-volume and type of ICU, the ICU size (OR = 1.50 (95% CI, 1.45 to 1.95), for 11 to 20 beds; OR = 2.02 (1.40 to 2.92), for >20 beds) and ≥2 clinical protocols (OR = 0.65 (0.42 to 0.99)) were the organizational characteristics associated with mortality.

## Conclusion

In a large sample of Brazilian ICUs, the implementation of clinical protocols was associated with better outcomes. Conversely, mortality was higher in larger ICUs.

## Acknowledgements

Funded by IDOR, CNPq and FAPERJ. Endorsed by BRICNet.

